# Lung Function Assessment as an Early Biomonitor of Mercury-Induced Health Disorders in Artisanal and Small-Scale Gold Mining Areas in Indonesia

**DOI:** 10.3390/ijerph15112480

**Published:** 2018-11-07

**Authors:** Sri Manovita Pateda, Masayuki Sakakibara, Koichiro Sera

**Affiliations:** 1 Graduate School of Science & Engineering, Ehime University, 2-5 Bunkyo-cho, Matsuyama City, Ehime Prefecture 790-8577, Japan; sakaki@chikyu.ac.jp; 2 Public Health Department, Faculty of Sport and Health, State University of Gorontalo, Jenderal Sudirman Street 6, Gorontalo City, Gorontalo Province 96100, Indonesia; 3 Faculty of Collaborative Regional Innovation, Ehime University, 3 Bunkyo-cho, Matsuyama City, Ehime Prefecture 790-8577, Japan; 4 Research Institute for Humanity and Nature, 457-4 Motoyama, Kamigamo, Kita-ku, Kyoto 603-8047, Japan; 5 Cyclotron Research Center, Iwate Medical University, 348-58 Tomegamori, Takizawa, Iwate 020-0173, Japan; ksera@iwate-med.ac.jp

**Keywords:** mercury, biomonitor, ASGM, spirometry, Gorontalo

## Abstract

The evaluation of mercury impact on humans is currently nonspecific because the body characteristics (homeostasis) of each human being varies. Therefore, in the early diagnosis of mercury toxicity, one of the most important monitoring parameters is the respiratory function examination. In this study, respiratory function was examined with a portable spirometer and correlated with the mercury levels in hair from the noses and heads of subjects. Samples were taken from artisanal and small-scale gold mining (ASGM) areas (villages of East Tulabolo and Dunggilata) and control areas (villages of Bongo and Longalo) in Gorontalo Province, Indonesia. A statistical analysis with the Mann–Whitney test (alternative) showed significant differences in lung function between the polluted and control areas (*α* = 0.03). The analysis of nasal and head hair samples with particle-induced *X*-ray emissions (PIXE) showed that the mercury levels in the ASGM area were considerably higher than in the more homogeneous control areas. This study confirms that a pulmonary function test is a quick and precise alternative way to monitor the impact of mercury on humans, especially atmospheric mercury, because we detected a negative correlation between pulmonary function and the level of mercury in hair.

## 1. Introduction

### 1.1. Background

Mining-associated problems in artisanal and small-scale gold mining (ASGM) areas have not been resolved. Economic problems are thought to be the main cause of the unresolved issues in these areas [1,2,3]. This is consistent with the increasing levels of pollution caused by mining activities [4]. Mercury is one of many health issues related to ASGM [5,6,7,8,9,10] because ASGM is the world’s largest user of mercury and a major source of anthropogenic contamination of the environment [11,12,13]. 

The effects of mercury on the human body generate a wide variety of symptoms and manifestations [8,10,14,15,16,17,18,19,20,21,22,23]. In the history of mercury effects on humans, the term “mad as a hatter” in the 1800s was attached to workers who had long-term exposure to mercury vapors, and suffered severe neurological disorders (mad hatter disease or erethism mercurials) [24]. The severe effects of mercury on the neurological system occured around Minamata Bay in Japan, known as Minamata disease [25]. Ha et al. (2016) reported in their paper that the effects of mercury are very widespread, not only in children and adults, but that the fetus has been exposed to mercury toxicity [14], and also reported by several studies [5,21,26,27,28,29,30]. However, no criteria for the diagnosis of mercury poisoning have been defined because there is no clear correlation between the clinical symptoms of intoxication and the level of mercury in the body [21,31,32].

Until now, we have focused only on systemic conditions of the human body, in which mercury is oxidized to Hg complex and dissolved in body fluids. However, this is a secondary effect, and we have ignored the primary effect in the organ systems that first come into contact with mercury pollutants [33]. One important system is the respiratory organs, which mediate the entry of mercury vapor into the body. Mining activities not only produce harmful mercury vapor to the respiratory system; stone-breaking and excavation processes also generate dust, which can cause pneumoconiosis [33]. This lowers the resistance of the respiratory system, which is then more susceptible to infectious diseases such as pneumonitis, respiratory distress syndrome, and even death [28]. The source of mercury that needs to be our concern together is atmospheric mercury, which turns out not only to expose people living around mining but also people who live far from mining areas [15]. For this reason, the examination of the respiratory system becomes an urgent aspect that needs to be an early biomonitor to prevent any significant impact.

Because the most frequently used diagnostic tools measure widely diverse parameters (such as hair, urine, and blood) [5], it is necessary to develop a single biomonitoring tool to estimate the prevalence of mercury effects on human health. Examination of the respiratory system would be one of the most accessible ways to monitor the effects of mercury on human health.

In this paper, we present data from an analysis of human respiratory function, which we compared with themercury levels in human hair. Our aims were: (1) to characterize the respiratory health status of the respondents; (2) to analyze the differences in the spirometry test results (describing lung function) between the respondents in the control and ASGM areas; and (3) to correlate the mercury content in hair samples with respiratory function.

### 1.2. Study Area and Selected Population

#### 1.2.1. Study Area

**Figure 1:**
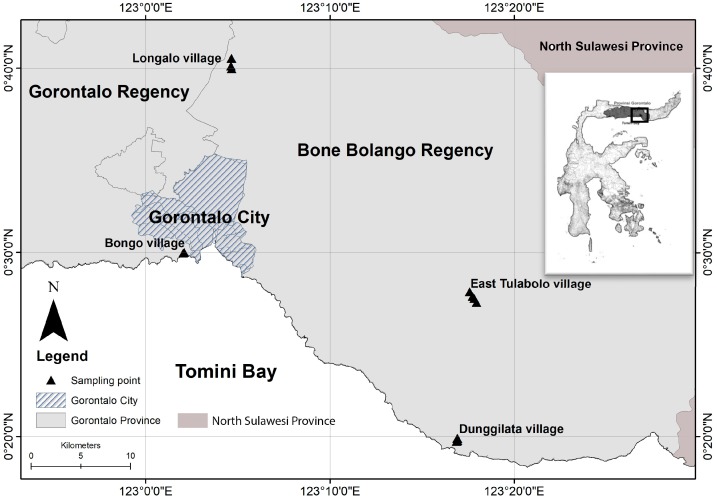
Location of sample points from four areas in two Regencies (triangle sign), in Gorontalo Province, Indonesia, wherein three areas located in Bone Bolango Regency (East Tulabolo, Dunggilata, and Longalo villages) and one area located in Gorontalo Regency (Bongo village).

Data were collected from four areas, two representing mining sites (ASGM area) and two control areas in Gorontalo Province, Indonesia. The first mining area was located in the mountainous area of the East Tulabolo village, East Suwawa District, Bone Bolango Regency. The second mining area was located in Dunggilata village, Bulawa District, Bone Bolango Regency, which is a coastal area on Tomini Bay, Sulawesi Island, as shown in Figure 1. The control data were collected from two villages: Longalo village, Bulango Utara District, Bone Bolango Regency; and Bongo village, Batudaa Pantai District, Gorontalo Regency. These two villages were selected as the control areas because they corresponded geographically to the mining areas, in that one was a mountainous area (Longalo village) and the other a coastal area (Bongo village). Therefore, it was also possible to homogenize the study populations.

#### 1.2.2. Population and Samples

Individuals whose health was checked are referred to as ‘respondents’, and the hair material taken from them is referred to as ‘samples’. The respondents who were sampled in this study were taken by accidental sampling. The inclusion criteria were: (a) registered as a permanent resident at the point of sampling; (b) willingness to be a subject of research; and (c) no moderate to severe systemic disease.

The selection of samples in the ASGM areas was not limited to miners because we wished to provide a broader picture of the effects of mercury in the community. Preference was given to respondents who were family members of miners because some miner families participate in the mining or periodically reside in these fields. The sample selection in the control areas was slightly tighter. The selected subjects had absolutely no contact with mining activities, whether as miners or in other work undertaken in the mining area, such as selling food or daily necessities. The respondents in the control areas were also selected based on the predefined inclusion criteria (above).

According to the ethical guidelines on conducting human-subject research, informed consent is very substantial. Before we obtained approval from the respondents, the study and their roles in it were explained to them. Approval was only obtained after a detailed explanation was given, which included: (1) the research background; (2) research purposes; (3) benefits of the research; (4) descriptions of the examination process and risks; and (5) assurance of the anonymity of all respondents.

## 2. Materials and Methods

The study began with a preliminary survey conducted in two mining areas in Gorontalo Province, located in the villages of East Tulabolo and Dunggilata, in 2017. After informed consent was given by the respondents, we collected their demographic data. These are supplemented by the characteristic information of the respondents as the analyzed variables.

### 2.1. Characteristics of Respondents

The direct interview was one of the methods used to collect the initial data of the respondents. The questionnaire contained a general data section, which included the respondent’s occupation. Information on the relationships of the respondents with mercury were important variables, and the items included: (i) the distance of residence from the mining area and the length of stay at that place; (ii) whether he/she worked in the mining area and the length of time in that job; (iii) whether he/she had ever worked as a miner, for what period, and in which work section. The final datum was the length of the period of exposure to mercury pollutants from the ASGM area.

### 2.2. General Health Assessment

A standard medical examination was performed to assess the respondent’s health, which included anamnesis, vital statistics, a physical examination, and supporting inspection. This examination was conducted by a trained nurse.

Anamnesis was performed by asking about the respondent’s current complaints and previous medical history, and also included a question about smoking history. Respondents who had smoked in the preceding 10 years were classified as ‘smokers’, and those who had never smoked or had quit 10 years earlier were classified as ‘nonsmokers’. Smoking status was calculated with the Brinkman Index (BI) based on the guideline from The Indonesia Society of Respirology [34], using the formula:

Brinkman Index = number of years of smoking × number of cigarettes smoked per day(1)

The Brinkman Index divides smokers into three categories: (1) heavy smokers, BI ≥ 600; (2) moderate smokers, BI = 200 − 599; and (3) mild smokers, BI ≤ 199.

The physical examination specifically addressed the respiratory and neurological systems. Height and weight were measured during the same examination. Smoking status, height, and weight were the basic data used in the spirometry analysis.

Spirometers were used for the lung function tests. Meanwhile, scalp and nose hair samples were collected for the laboratory analysis of mercury levels. Nasal hair sampling was performed manually or with tweezers. The scalp and nose hair samples were analyzed at the Cyclotron Research Center, Iwate Medical University, Japan.

### 2.3. Spirometry Test

Spirometry is a useful basic tool in the diagnosis of lung disease. It is used to assess respiratory function, primarily by evaluating the breathing pattern in pulmonary fibrosis or asthma, and is also very useful in health surveillance. In this study, we used the simplest hand-held portable spirometer, which was easy to carry to the points of sampling. This tool is supported by computer software for spirometry [35], so the final results can be obtained directly and quickly.

The use of the spirometers was in accordance with the guidelines of the American Thoracic Society Standards, 2005 [36]. All procedures were performed based on these standards, including the preparation of the patient and tools, the process of assessment, and the interpretation of the results. Preparing the person for examination included providing a comfortable location for the respondent, a chair that allowed their feet to touch the floor, and prepared drinking water. The tools were calibrated with the syringe as the part of the built-in instrument of the spirometer. Some requirements to be met by respondents were: (1) no alcohol consumption in approximately 4 h before the test; (2) had not recently consumed a heavy meal; (3) no tight clothing; and (4) no heavy physical exercise in the preceding 30 min. The data required by the spirometry software were the identification number, name, age, sex, ethnic group (we selected ‘not defined’), address, height, weight, smoking status, and other general information on the medical history of the respondent. Importantly, we explained the purpose and the correct technique of the test to each respondent. The spirometer consists of a turbine (in this study, we used a disposable turbine flowmeter), nose clip, and the spirometer. The examination was performed with three repetitions maximally unless the initial examination gave normal results. The results were read directly on the computer software.

In this study, we considered the forced vital capacity (FVC), the forced expiratory volume in 1 s (FEV1), and the overall interpretation of the spirometry test. The software data showed the predicted values for FVC and FEV1, the test results, the percentage differences between the test results and the predicted values, and the final interpretation. The predictions of FVC and FEV1 were based on the standards of the American Thoracic Society equations for the Japan Respiratory Society. Because the ethnic group options available did not include ‘Indonesian’, we selected ‘not defined’, and the equations used to predict these values chosen were those of the Japan Society, based on the similarities in Asian characteristics.

### 2.4. Nose and Scalp Hair Samples

Nose and scalp hair was sampled from each respondent, except under special circumstances. For instance, some respondents (usually male) had very short hair or were virtually bald, so their scalp hair was not sampled; and some respondents (usually female) declined to have their nose hair sampled when this proved hard. The samples were then stored by wrapping them in weighing paper and labeled.

The nose and scalp hairs were analyzed with *X*-ray fluorescence named particle-induced *X*-ray emission (PIXE). This analysis used proton beam energies of 2.5–3 MeV. The uniform density beam was collimated to a diameter of 6 mm and used a combination of thick nickel foil and a diffuser in a graphite collimator system. The sample was kept in rectangular targets and placed at an angle of about 35° to the horizontal proton beam axis. The *X*-ray emissions from the sample were detected with a Si (Li) detector and passed through the target chamber. A 300-μm-thick Mylar absorber was used to reduce the low energy *X*-rays that were produced [16]. The PIXE analysis was done at the Cyclotron Research Center, Iwate Medical University, Japan, after the samples underwent several different stages of preparation. Hair samples should go through several processes before being ready to send for PIXE analysis. This process is divided into three stages, that is: Stage 1. pre-washing; Stage 2. washing; and Stage 3. post-washing. The washing process aims to remove contaminants, like dust, dirt, bacteria, and other possible elements.

1. Pre-washing stage
  Hair samples must be selected with a hair length greater than 2.5 cm and not excessively lean. We must pick out the part that is closer to the hair root.
2. Washing stage
  The hair samples were put into a beaker glass filled with Milli-Q water (18.2 MΩ·cm), and were then washed in an ultrasonic cleaner bath (AS ONE corporation, Osaka, Japan) for 5 min. Afterwards, the samples were dried on sterile paper towels at room temperature. After drying, the samples were washed by stirring in acetone (Wako Pure Chemical Industries, Ltd., Osaka, Japan) for 5 min and washed again with Milli-Q water, then dried on sterile paper towels at room temperature.
3. Post-washing stage
  The eight strands of dried hair samples were attached in parallel to the midway position of the sample holder. Sample labels were written on the side part of the holder.

### 2.5. Analytical Method

In this cross-sectional study, 159 respondents were included, and after the inclusion criteria were applied, 133 respondents remained, of which 27, 31, 35, and 40 were from East Tulabolo, Dunggilata, Longalo, and Bongo, respectively. The data were analyzed statistically with a computerized spreadsheet and statistical software. The main variables examined in the study were the spirometry results, and the comparative variables were the two types of sampling areas, the ASGM and control areas. The complementary variables were smoking status and occupation (miner or non-miner). We used a *χ*^2^ test to compare the spirometry results in the different areas, with smoking status as a classifier. We used an unpaired *t*-test or the Mann–Whitney test for the comparative analyses. The significance value was 95% with *α* = 0.05.

## 3. Results

### 3.1. Characteristics of Respondents

Samples were collected from four areas, three in the Bone Bolango Regency and one in the Gorontalo Regency. Samples were collected from 133 respondents, 58 from ASGM areas and 75 from control areas, of whom 54% were male and 46% female.

The characteristics of the respondents (age, sex, and body mass index [BMI]) are shown in Table 1. The mean age of the respondents was 40.7 ± 2.61 years and the majority were aged >35 years. The mean BMI was normal at 23.1 ± 0.8, with a median value of 21.1 (also normal). This indicates that the respondents’ data were in the normal ranges.

**Figure 2:**
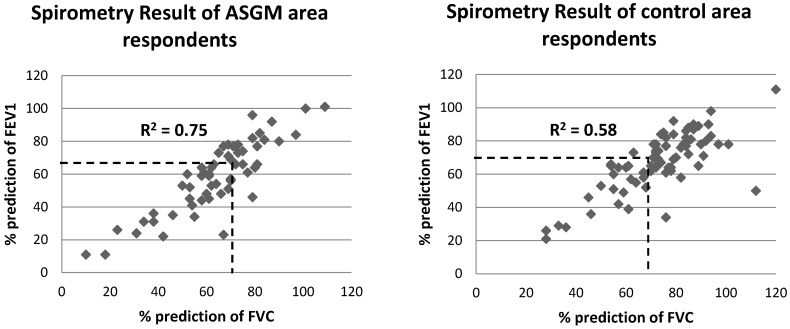
Correlation between Force Vital Capacity (FVC) and Forced Expiratory Volume in 1 s (FEV1) in ASGM (artisanal and small-scale gold mining) and control areas. Area laid below the dotted line express the abnormal results of FVC and FEV1 representing the interpretation of spirometry test result.

Figure 2 shows the data describing the relationships between the respondents and their mercury exposure by occupation. In the East Tulabolo village, 44% of the respondents worked as miners, and 32% in Dunggilata village, thus 56% and 68% of the respondents did not work as miners in these villages, respectively. In total, of the 58 respondents (in the ASGM area), 38% were miners and 62% were non-miners.

Table 2 shows the spirometry results and their determinants, including smoking status, lung condition (FVC and FEV1 measured by spirometry), and the interpretation of the spirometry results for each sampling area. In total, 80 respondents were smokers, but on average only mild smokers. Nevertheless, moderate and heavy smokers also accounted for a considerable number of smokers overall.

The highest average FVC score was 2.67 ± 0.13 L (Bongo village) and the lowest was 2.13 ± 0.16 L (Dunggilata village). The highest FEV1 value was in the Bongo village (2.19 ± 0.13 L) and the lowest was in the Dunggilata village (1.69 ± 0.15). The average values for the percentage difference between the test results and the predicted values of FVC and FEV1 in the ASGM areas were low, based on the American Thoracic Society Standard (normal is >70%), although the average value for FEV1 was only a little lower than the normal value. This is shown in detail in Figure 2.

Interpretation of the spirometry data showed that fewer than 50% of the respondents in the ASGM areas had normal results (29.6% and 41.9%), whereas, in the control areas, more than 50% of the respondents had normal results (57.1% and 62.5%).

### 3.2. Analysis of Lung Function

We used two approaches in the statistical analyses. In the first, we examined the association between smoking grade and the spirometry test results as the effect factor, by area. In the second approach, we compared the results of the spirometry tests according to geographic location and occupation as a miner in an ASGM area.

The spirometry measurements were made in the four sampling areas over 2–3 days in each area. Explaining the examination process and technique to each respondent required considerable time.

Repetitions were performed for each respondent, and if the respondent’s technique was inadequate, he/she was asked to rest for approximately 15 min and the test was repeated. During the waiting period, the respondent was coached in the correct technique by a trained nurse.

Figure 2 explained that the area beneath the dashed line is the area of abnormal FVC and FEV1 values (<70%). The point value for ASGM areas, mostly in this area compared to the control area, it appears that the density of point value is in the abnormal area. 

The spirometry results are more clearly described in Table 3, according to geographic location and occupation as a miner in an ASGM area. The statistical analysis was performed with an unpaired *t*-test or the Mann–Whitney test. The point samples were categorized geographically into ASGM and control areas. An important distinction was made between miners and non-miners in the ASGM areas. A comparison of the three classifications of spirometry results (based on area) showed a significant value of 0.03 (*p* < 0.05). 

A multivariate analysis was applied to four variables, that is: (1) respondent area; (2) smoking grading; (3) occupation; and (4) duration of work, and just two variables passed the simple logistic regression, number 1 and 2. The final result concluded that smoking grade had a high probability, but acts as a confounder variable (*p* value = 0.124), and respondent area was a significant variable correlated with inhabitant lung function.

### 3.3. The Relationship between Mercury Levels in Head and Nose Hair with Spirometry Test Results

**Figure 3:**
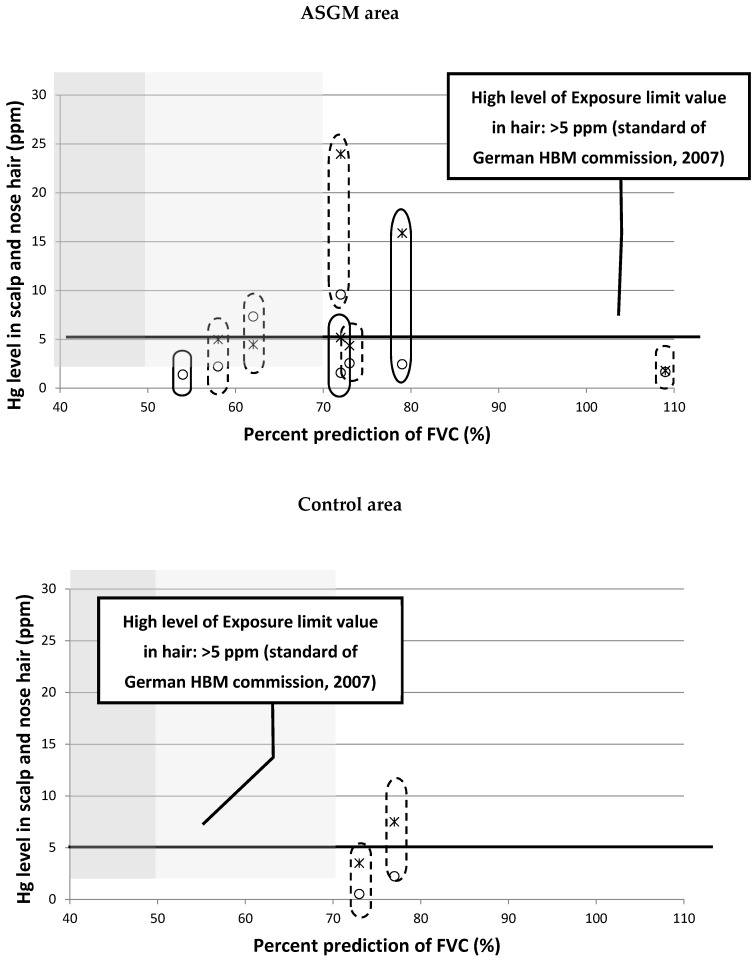
Level of mercury in: 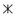 nose hair (PIXE);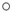 scalp hair (PIXE). Lung function by spirometry test: 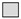 severe disorder; 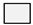 middle disorder; 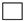 normal 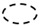 mild to no smoker 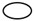 moderate smoker. PIXE: particle-induced X-ray emissions.

Nineteen samples, including nine nose hair samples and 10 scalp hair samples, were analyzed with PIXE. Seven samples of nose hair and eight samples of scalp hair were from the ASGM areas and four samples (nose and scalp hair) were from the control areas, as shown in Figure 3.

Based on the German Human Biomonitoring (HBM) Commission standard (2007) [37], the level of mercury in hair is defined as high at >5 ppm (referred to as HBM II). The safe level is considered to be in the range ≤1 ppm (referred to as HBM I). Mercury levels between HBM I and HBM II are considered warning levels [38]. The mercury levels in the hair samples are shown in Figure 3. Most samples were above HBM I, even in the control areas. The highest level was 24.4 ppm and the lowest was 0.52 ppm. In all other samples, the mercury level exceeded 1 ppm.

An interesting pattern was observed when the mercury levels in hair were correlated with the spirometry results (in this correlation, we used the percentage prediction of FVC to represent the lung capacity). Our hypothesis was that higher levels of mercury in the hair, which represents greater exposure to mercury, would correlate positively with greater respiratory dysfunction. However, Figure 3 shows the opposite (negative) correlation. Respondents with high levels of mercury in their hair actually showed normal lung function. We also tried to include the smoking grading in this graphical analysis and correlation results also denied our hypothesis.

## 4. Discussion

Because the specific effects of mercury contamination in humans are diverse and nonspecific, there is something more important to deal with than to find that particularity—in other words, another measure of toxicity is required. However, the resilience of each individual is different because it is influenced by many factors, including age, sex, ethnicity, and so forth. Nevertheless, this homeostasis is easily disturbed by pollutants, depending on their dose and toxicity. Chronic toxicity usually involves low-dose and/or discontinuous exposure to the toxicant. In the course of the corresponding disease, this is called the ‘latent period’ and is a common characteristic of diseases related to geological toxicity [20].

This phenomenon is shown in Figure 3, in which the increase in hair mercury levels did not correlate with the lung function status, which was expected to deteriorate with increased exposure to mercury. Of the 10 respondents whose hair was analyzed, seven were miners and three non-miners. These data indicate that there is no clear correlation between the clinical symptom of exposure and the level of mercury in the body, which implies that there are no defined criteria for the diagnosis of mercury poisoning [21].

The exposure of the respiratory system to mercury vapor, especially in miners, should be of special interest because these pollutants can also affect the surrounding communities indirectly [1], usually as chronic toxicity [20]. This is demonstrated in the statistical analysis of our spirometry results in miners and non-miners in the ASGM areas, as shown in Table 3, where the difference was not significant (Mann–Whitney test, *α* = 0.63). However, the spirometry results differed significantly (Mann–Whitney test, *α* = 0.03) between the ASGM areas and control areas when the whole sample was analyzed.

## 5. Conclusions

In this study, the formulated hypothesis is contradicting the research results. The correlation between mercury levels in hair should be in line with the respiratory system disorder, but those presented to be negative correlations, as shown in Figure 3, and multivariate analysis reinforced significant differences between ASGM and control areas. The effects of mercury as a toxic material directly in contact with the human respiration system means that initial monitoring is a matter of priority. We concluded that monitoring the toxic effects of mercury on human health is essential for early diagnosis so that severe consequences can be avoided.

Human health parameters and their surveillance are not only important at the level of individuals, as community-level surveillance can accommodate the health differences present in all human beings, even under the same circumstances. The statistical correlation showed a significant number of differences in respiration function of respondents between ASGM and control areas (*p* = 0.03 with SI 95%). An individual community must be seen as part of a whole community, so that community indicators must be our common concern.

## Figures and Tables

**Table 1:** Characteristics of respondents by artisanal and small-scale gold mining (ASGM) and control areas.

Characteristics	ASGM Area		Control Area		Mean
	East Tulabolo	Dunggilata	Longalo	Bongo	
*n* (respondents)	27	31	35	40	133
Age (years old)					
• Modus	21	43	46	32	35,5
• Mean (SD)	37.1 (2.91)	41.7 (3.08)	45.8 (2.70)	38.2 (1.74)	40.7 (2.61)
• Median	35	43	46	36	40
Sex (%)					
• Male	51.9	58	43	63	54
• Female	48.1	42	57	37	46
Body Mass Index (kg/m^2^)					
• Mean (SD)	22.6 (0.75)	21.9 (1.08)	22.7 (0.77)	25.3 (0.74)	23.1 (0.80)
• Modus	23.2	21.6	19.8	19.7	21.1
Work as a miner (%)					
• Yes	44	32	0	0	19
• No	56	68	100	100	81

*n* = number of respondents; SD = Standard Deviation.

**Table 2:** Respiratory assessment result by smoking grade, Forced Vital Capacity (FVC), Forced Expiration Volume in 1 s (FEV1), and spirometry interpretation.

Respiratory Assessment	ASGM Area		Control Area	
	East Tulabolo	Dunggilata	Longalo	Bongo
*n* (respondents)	27	31	35	40
Smoking Status (*n*)				
• Yes	13	16	25	26
• No	14	15	10	14
Smoking Grade (%)				
• Mild	25.9	19.4	17.1	25.0
• Moderate	18.5	9.7	11.4	10.0
• Heavy	3.7	19.4	0.0	0.0
FVC (Liter)				
• Mean (SD)	2.32 (0.14)	2.13 (0.16)	2.29 (0.14)	2.67 (0.13)
• Modus	2.57	1.54	1.58	2.15
% prediction of FVC				
• Mean (SD)	67.0 (18.0)	61.8 (19.9)	74.6 (19.4)	72.0 (15.1)
• Modus	58	70	73	72
FEV1 (Liter)				
• Mean (SD)	1.86 (0.14)	1.69 (0.15)	1.82 (0.12)	2.19 (0.13)
• Modus	2.07	0.89	0.68	1.79
% prediction of FEV1				
• Mean (SD)	61.8 (20.2)	56.4 (22.6)	68.0 (17.3)	67.1 (17.6)
• Modus	66	31	64	78
Spirometry Interpretation (%)				
• Normal	29.6	41.9	57.1	62.5
• Middle	44.4	25.8	28.6	27.5
• Severe	25.9	32.3	14.3	10.0

*n* = number of respondents; SD = Standard Deviation. Note: Percentages may not add up to exactely 100%, owing to the rounding off.

**Table 3:** Analysis of spirometry test results based on area, smoking grade, occupation of respondent in ASGM area, and duration of work as a miner.

Classification	*n*	Spirometry Test Results (%)			*p*
		Normal	Middle	Severe	
Based on Area					0.03 ^0^
― ASGM area	58	36.2	34.5	29.3	
― Control area	75	60.0	28.0	12.0	
Smoking Grade				
― Mild	29	55.2	27.6	17.2	0.04 ^0^
― Moderate	16	37.5	37.5	25.0	
― Heavy	7	14.3	14.3	71.4	
Occupation in ASGM area					0.63 ^0^
― Miner	22	36.4	27.3	36.4	
― Non-miner	36	36.1	38.9	25.0	
Duration of work (as a miner)					0.69 * r = −0.07
― Acute (<5 years)	8	25.0	33.3	50.0	
― Chronic (≥5 years)	14	75.0	66.7	50.0	

*n* = number of respondents; *p* = significant value (*p* < 0.05); ^0^ Mann–Whitney test; * Somers’ D test.
